# Editorial: Foodborne Pathogens: Hygiene and Safety

**DOI:** 10.3389/fmicb.2019.01974

**Published:** 2019-08-27

**Authors:** Maria Schirone, Pierina Visciano, Rosanna Tofalo, Giovanna Suzzi

**Affiliations:** Faculty of Bioscience and Technology for Food, Agriculture and Environment, University of Teramo, Teramo, Italy

**Keywords:** food, microorganisms, virulence, illness, preservatives

## Introduction

The foodborne outbreaks occurred in last decades highlight the importance of the development and implementation of preventive measures and programs aiming at ensuring food safety on one hand and constituting a common basis for the hygienic production of food on the other hand. In particular, a *farm to fork approach* has been applied in all sectors of food production chain in order to improve hygiene and reduce all potential biological hazards. The food supply chain is very complex because of the differences in food composition and processing and this can result in emergence and re-emergence of foodborne pathogens. However, many factors related to an increase in foodborne illness have been reported, such as the change in eating habits and consumer preferences, increased international travels, change in food processing, production and distribution, pathogen adaptation to new environments, acquisition of virulence factors and antimicrobial drug resistance by microorganisms, advances in pathogen detection methods, inadequate sanitation and vector control measures, inadequate public health services, including consumer information (Smith and Fratamico, 2018). This Research Topic titled “Foodborne Pathogens: Hygiene and Safety” focuses on important food safety concerns such as the potential presence of pathogens in food as well as their toxins/metabolites, the resistance to antibiotics or sanitizers, and other virulence characteristics. It includes four reviews and 44 original research papers. The main foodborne pathogens studied herein are: *Campylobacter jejuni, Cronobacter sakazakii, Escherichia coli, Listeria monocytogenes, Salmonella* spp., and *Staphyloccus aureus*, but some other researches deal with *Helicobacter pilori, Klebsiella pneumoniae, Vibrio parahaemolyticus*, mycobacteria, and molds as well. Studies on characterization and genetic typing of foodborne pathogens, detection methods and inactivation of these microorganisms by natural preservatives derived from plant sources, essential oils and biocontrol, and influence of probiotics are also reported.

## Prevalence and Monitoring of Pathogens in Food

Foodborne diseases represent one of the most important public health troubles worldwide. The potential of foodborne pathogens to cause illness or even death in consumers highlights the importance of such events and consequent need of their monitoring and prevention. Millions of cases of foodborne illnesses and/or chronic complications are reported in many countries every year (Heredia and García, 2018). Li S. et al. studied the prevalence and characteristics of Non-typhoidal *Salmonella* isolated from poultry meat (broilers and spent hens) from supermarkets in China. Three serotypes were identified in 40 *Salmonella* strains and *Salmonella* Enteritidis resulted as dominant. The antibiotic resistance was tested as well, showing the highest rates to ampicillin for the strains isolated from commercial broilers, and to nalidixic acid for those isolated from spent hence. Thung et al. investigated the prevalence of *Salmonella* spp. in different beef meat samples from retail markets in Malaysia as well as the virulence genes and antimicrobial resistance. Eight different serovars were identified and *Salmonella* Agona was the predominant one. All 23 isolates were resistant at least to three antibiotics. Colello et al. determined the prevalence of *Salmonella* spp. in 764 samples collected from swine farms, slaughterhouses, boning rooms, and retail markets. The strains were classified into five serotypes (i.e., *Salmonella* Typhimurium, *Salmonella* Kentucky, *Salmonella* Brandenburg, *Salmonella* Livingstone, and *Salmonella* Agona) and showed different resistance to antibiotics.

The microbiological quality (mesophilic aerobic bacteria, total coliforms, yeasts, and molds) and safety level (*E. coli* O157:H7, Shiga toxin-producing *E. coli, Salmonella* Enteritidis, *Salmonella* Typhimurium, *Listeria* spp., and *L. monocytogenes*) of organic and conventional vegetables from Malaysia were evaluated. *Salmonella* spp., *L. monocytogenes*, and *Listeria* spp. were the most representatives, with no trend between organically or conventionally grown vegetables (Kuan et al.). The presence of total and pathogenic *V. parahaemolyticus* strains was detected in short mackerel samples collected from different retail markets in Malaysia. The antimicrobial susceptibility profiles were also studied, showing a resistance to penicillin G and ampicillin (Tan et al.).

The genetic diversity as well as the antibiotic resistance and biofilm formation of *Cronobacter* spp. recovered from spices and cereals were studied by Li Y. et al. *Cronobacter sakazakii* was the most common species, and 62.5% of 40 *Cr. sakazakii* strains were non-biofilm producers. Parra-Flores et al. evaluated the presence of *Cr. sakazakii*, microbiological levels of aerobic plate count and *Enterobacteriaceae* in dairy product batches associated with a recent food alert in Chile.

Sevilla et al. investigated the presence of members of the genus *Mycobacterium* by culture and PCR-based methods in raw dairy and meat products purchased at different supermarkets in Spain. Mycobacterial DNA was detected in 23 out of 257 samples, corresponding to *Mycobacterium avium, Mycobacterium tuberculosis*, and other non-tuberculous mycobacteria.

Wang W et al. submitted two papers to this Research Topic, the first one concerned the complete genomic analysis of a *Salmonella* Typhimurium isolate from ready-to-eat pork samples in China, the second dealt with the prevalence of *S. aureus* among raw milk from dairy cows with clinical mastitis.

Lipophilic marine biotoxins belonging to okadaic acid, pectenotoxin, yessotoxin, and azaspiracid groups were determined in specimens of mussels collected along the coasts of the Central Adriatic Sea (Italy) by LC-MS/MS. The concentrations exceeded the maximum regulatory limits only for 11 out of 400 samples, and some samples showed a multi-toxin mixture contamination (Schirone et al.).

## Antimicrobial Resistance and Virulence Factors

Microbial interactions can show beneficial or detrimental effects that influence the fate of pathogenic species contaminating foods. The study of such interactions can provide a new knowledge about the different activities of the microorganisms from proliferation and metabolism to pathogenicity and virulence (Zilelidou and Skandamis, 2018).

Dairy products can host microorganisms belonging to *Enterobacteriaceae* family showing multidrug resistance to antibiotics and other virulence factors such as production of biofilm and synthesis of proteolytic and lipolytic enzymes responsible for spoilage. Their presence can be reduced or avoided through good hygiene conditions during processing and manufacturing, as well as storage and distribution (Amorim et al.). Chagnot et al. investigated the adhesion of *E. coli* O157:H7 to well-defined types of skeletal muscle and demonstrated that such microorganism mainly adhered to the extracellular matrix of muscle cells, with no significant differences among the different constituent myofibres, whereas the influence of post-mortem structural modifications of muscle tissues was substantial.

The adhesion capacity of 40 *C. jejuni* strains to abiotic surfaces was studied. All *C*. *jejuni* strains were shown to be capable of forming strong biofilms when Mueller Hinton medium was supplemented with chicken juice. However, the use of biocides was effective in controlling viable cells of strains in biofilm (Melo et al.). Oh et al. demonstrated that ferrous and ferric iron stimulated biofilm formation in *C. jejuni* through oxidative stress. Premarathne et al. determined the prevalence and antibiotic resistance of *Campylobacter* spp. in the beef food system in Malaysia. Most isolates were identified as *C. jejuni*, with a high percentage resistant to tetracycline and ampicillin.

The effect of cold stress on the adhesion to abiotic surfaces and biofilm formation of 22 *L. monocytogenes* strains from different serogroups and origins was studied by Lee et al. Such study demonstrated the increase of the adhesion capacity, whereas the cold-adapted cells remained in planktonic form. Pasquali et al. studied the persistence and physiological adaptation to food-processing environmental stress of *L. monocytogenes* strains from a rabbit meat processing plant. While some strains showed a resistance to sanitizers, some others were biofilm producers and these specific characteristics could contribute to their high prevalence. The nucleotide diversity of *L. monocytogenes* strains from human clinical cases—as well as food or food-related environments originating from three different geographical locations (i.e., Australia, Greece, and Ireland)—was studied by Poimenidou et al. The authors demonstrated that virulence genes showed different evolutionary pathways affected by the origin and serotype of the specific strain.

Lang et al. demonstrated that drying of milk powder increased the Caco-2 cell invasion capacity of two pathogens, i.e., *Salmonella enterica* and *Cr. sakazakii*, probably due to the activation of stress response transcriptional factors, and a subsequent heat treatment did not offset the loss of cultivability that was observed in the experimental design.

Javed et al. described the characteristics, prevalence, survival, and transmission, as well as pathogenesis and virulence determinants of *Helicobacter pullorum*. Such microorganism causes gastroenteritis in poultry, but it is also an emerging zoonotic bacterium associated with enteric infections in humans with colitis, hepatitis, and recurrent diarrhea.

## Detection Methods Applicable in Food Industry

A microfluid system combining loop-mediated isothermal amplification with gold nanoparticles for rapid detection of *Salmonella* spp. in food samples was performed. Such method showed relative sensitivity, specificity and accuracy of 100% and could be used in the food industry as a simple, inexpensive and fast analytical approach (Garrido-Maestu et al.). A new standard operating procedure for multiple-locus variable number tandem repeat analysis (MLVA) of *Salmonella* Dublin was proposed by Vignaud et al. The MLVA scheme was applied to a foodborne outbreak occurred in France in 2012, in order to discriminate between epidemiologically related strains and sporadic case strains. Fong et al. characterized four *Salmonella* phages isolated from irrigation water, cattle feces, and sediment from irrigation ditches, based on their phenotypic and genotypic determinants, and assessed their infectivity against various *Salmonella* strains *in vitro*. Among them, the phage isolate SI1 was the most effective in control of *Salmonella* Enteritidis in sprouting alfalfa seeds artificially contaminated.

The study of Ogrodzki and Forsythe described the application of three genotyping methods (Multilocus Sequence Typing, MLST, capsular profiling of the *K*-antigen and colanic acid byosinthesis regions and CRISPR-*cas* array profiling) to discriminate different species belonging to *Cronobacter* genus. Chase et al. found a *Cr. sakazakii* isolate, H322, in a batch of powdered infant formula (PIF) and two other isolates showing indistinguishable Pulsed Field Gel Electrophoresis patterns with H322, during routine testing of these products ready for distribution. Therefore, whole genome sequencing, as well as microarray analysis, was applied to these strains, showing a phylogenetic relation among them. This study confirmed that the pathogen could persist within the PIF manufacturing facility for years.

Wang J. et al. developed a novel approach to predict the growth kinetics of *S. aureus* on rice cake under different environmental conditions. These probability models could be useful for food safety management and microbiological risk assessment of such pathogen.

*Listeria monocytogenes* encodes a functional ArgR, a transcriptional regulator with specific functions in arginine metabolism regulation and acid tolerance. Cheng et al. showed that a single ArgR regulator could have opposite regulatory effects on the arginine deiminase pathway in an arginine-independent and dependent manner under neutral and acidic conditions, respectively.

Henri et al. compared different genomic methods, i.e., MLST, Whole Genome Sequencing (WGS), and Single Nucleotide Polymorphism (SNP), used to cluster *L. monocytogenes* strains. This study revealed high concordance between MLST and SNP approaches for diagnostic laboratories responsible for outbreak detection and surveillance.

Williams et al. described a rapid flow cytometric method for determining *E. coli* O157:H7 contamination in raw spinach. This method could be used as a screening tool to detect such microorganism in food. The presence of two distinct *loci* of heat resistance on a plasmid encoding type three fimbriae and three bacteriocins, in 1 out 90 *E. coli* raw milk cheese strains, was investigated. Such plasmid was transferable to other *E. coli* strains including Shiga-toxin-producing strains, posing great concern in food production environments (Boll et al.). Hussain et al. evaluated the contamination with pathogenic and/or multiresistant *E. coli* among broiler free-range chicken specimens (ceca and meat). The isolates were characterized using both conventional typing and WGS and compared with human *E. coli* pathotypes. The results showed that the poultry *E. coli* strains shared closer genetic identity to human *E. coli*. Zhang B. et al. demonstrated that a specific genetic marker (named fimbrial gene *z3276*) of Enterohemorrhagic *E. coli* O157:H7 encoded multifunctional structures with properties contributing to host colonization and bacterial survival in the environment.

The regulatory mechanism of secondary metabolism by comparative transcriptomic in *Aspergillus flavus* was studied by Yao et al. Such approach allowed the authors to identify known and novel regulators required for aflatoxins biosynthesis.

Zhang S. et al. determined biotypes, serotypes, virulence genes, and antimicrobial resistance patterns of *K. pneumoniae* strains from retail foods in China. The authors reported that some strains from the same geographic area had a closer relationship and they showed high levels of resistance to ampicillin.

Yang et al. utilized a proteomic approach involving anti-acetyl lysine-based enrichment and highly sensitive mass spectrometry to identify the global acetylated proteome and investigate lysine acetylation in *Trichinella spiralis*.

Zhao et al. described the surface enhanced Raman spectroscopy as testing technology used for the detection of pathogenic bacteria in food. Such method can be considered fast, simple, specific, and sensitive.

## Promising Strategies for Food Preservation

Preservation technologies are applied to extend the shelf-life, improve the hygienic quality, and ensure the safety of food. In food industry bacteriocins or other natural preservatives such as herbal extracts and essential oils are used as alternative to prevent the growth of both pathogenic and spoiling microorganisms (Martínez et al., 2019; Nazari et al., 2019).

Gray et al. described novel biocontrol methods such as bacteriophages, endolysins, bacteriocins, and plant derived products (essential oils) for the prevention of biofilm formation by *L. monocytogenes* in food production facilities. The inhibitory effect of *Hedychium spicatum* L. essential oil and radiation on production of deoxynivalenol and zearalenone by *Fusarium graminearum* in maize grains was studied by response surface methodology. The results showed a reduction of fungal growth rate as well as mycotoxin content (Kalagatur et al.).

Bajpai et al. described a significant antibacterial activity of a quinoline compound (jineol) isolated from the insect *Scolopendra subspinipes mutilans* against two selected foodborne pathogens (i.e. *E. coli* O157:H7 and *S. aureus* KCTC-1621). Such compound could be used as alternative means of antimicrobial in pharma and food industries.

The study of García and Cabo focused on the optimization of *E. coli* inactivation by a quaternary ammonium compound based on a mathematical model. The results showed that the optimal disinfectant dose increased exponentially with the initial bacterial concentration.

Different pressure-temperature combinations were applied to investigate the inactivation kinetics of *E. coli, Listeria innocua*, and *S. aureus* in black tiger shrimp. *Staphylococcus aureus* was the most baro-resistant species among the three bacteria. Such study could be used to predict non-linear survival curves of other microorganisms in foods (Kaur and Rao).

In their study, Kiran et al. isolated an actinobacterial strain from a marine sponge producing a lipopeptide that was demonstrated to be an effective emulsifier as well as good antioxidant and protective agent against *S. aureus*. The authors used this lipopeptide as food additive in muffin production with good results in organoleptic qualities of such food.

Kollanoor Johny et al. evaluated the antimicrobial effects of subinhibitory concentrations of two plant-derived compounds (i.e., *trans*-cinnamaldehyde and eugenol) on different genes of *S. enterica* serotype Enteritidis phage type 8 associated with virulence, colonization, motility, and invasion capability of such pathogen.

Mohanta et al. described the biological synthesis of silver nanoparticles using a cell-free aqueous leaf extract of plant *Protium serratum* and their antibacterial activity against some foodborne pathogens, i.e., *S. aureus, E. coli*, and *Pseudomonas aeruginosa*. The authors suggested the application of such nanoparticles in food packaging materials as well as disinfectant and cleaning agents.

Nair and Kollanoor Johny submitted two papers to the present Research Topic, the first study described the potential of pimenta leaf essential oil in reducing *Salmonella* Heidelberg attachment on to turkey skin during poultry processing, whereas the second work studied the antimicrobial function of a dairy-originated probiotic strain against multidrug resistant *Salmonella* Heidelberg in poults, i.e., young turkeys. The cecal colonization, dissemination to internal organs and potential for skeletal muscle deposition of multidrug resistant strains of *Salmonella* Heidelberg were studied by a challenge experimental design in poults and adult turkey hens. The results showed the highest recovery in the cecum followed by spleen, liver, thigh, and breast, and could be used to better control this microorganism at farm level and improve the safety of turkey products (Nair et al.).

## Conclusions

The high number of studies collected in this Research Topic confirms the importance of foodborne pathogens as a global issue and provides a robust and up-to-date scientific advice. It has been highlighted how much important and essential is a rapid detection of foodborne pathogens by sensitive culture independent methods and by new technologies such as WGS or other biomarkers assay analysis. The outbreak investigations play also key roles in the prevention of foodborne pathogens growth and diffusion, such as their food vehicles and how the contamination can occur in the food supply chain. The positive results of this Research Topic suggest to collect additional and new data for the future on this topic “Foodborne pathogens: hygiene and safety.”

## Author Contributions

MS and PV drafted the editorial. RT and GS contributed to editorial revision. All authors approved the final paper.

### Conflict of Interest Statement

The authors declare that the research was conducted in the absence of any commercial or financial relationships that could be construed as a potential conflict of interest.
